# A Comparative Nitrogen Balance and Productivity Analysis of Legume and Non-legume Supported Cropping Systems: The Potential Role of Biological Nitrogen Fixation

**DOI:** 10.3389/fpls.2016.01700

**Published:** 2016-11-21

**Authors:** Pietro P. M. Iannetta, Mark Young, Johann Bachinger, Göran Bergkvist, Jordi Doltra, Rafael J. Lopez-Bellido, Michele Monti, Valentini A. Pappa, Moritz Reckling, Cairistiona F. E. Topp, Robin L. Walker, Robert M. Rees, Christine A. Watson, Euan K. James, Geoffrey R. Squire, Graham S. Begg

**Affiliations:** ^1^Ecological Sciences, James Hutton InstituteDundee, UK; ^2^Leibniz-Centre for Agricultural Landscape Research, Institute of Land Use SystemsMüncheberg, Germany; ^3^Department of Crop Production Ecology, Swedish University of Agricultural SciencesUppsala, Sweden; ^4^Department of Agroecology and Environment, Aarhus UniversityTjele, Denmark; ^5^Cantabrian Agricultural Research and Training Centre, Government of CantabriaMuriedas, Spain; ^6^Eco-efficient Cropping Systems, University of CordobaCordoba, Spain; ^7^Department of Agriculture, Mediterranea University of reggio CalabriaReggio Calabria, Italy; ^8^Research Division, Scotland's Rural CollegeEdinburgh, UK; ^9^Department of Crop Science, Agricultural University of AthensAthens, Greece

**Keywords:** legumes, biological nitrogen fixation, crop rotation, nitrogen balance, productivity

## Abstract

The potential of biological nitrogen fixation (BNF) to provide sufficient N for production has encouraged re-appraisal of cropping systems that deploy legumes. It has been argued that legume-derived N can maintain productivity as an alternative to the application of mineral fertilizer, although few studies have systematically evaluated the effect of optimizing the balance between legumes and non N-fixing crops to optimize production. In addition, the shortage, or even absence in some regions, of measurements of BNF in crops and forages severely limits the ability to design and evaluate new legume–based agroecosystems. To provide an indication of the magnitude of BNF in European agriculture, a soil-surface N-balance approach was applied to historical data from 8 experimental cropping systems that compared legume and non-legume crop types (e.g., grains, forages and intercrops) across pedoclimatic regions of Europe. Mean BNF for different legume types ranged from 32 to 115 kg ha^−1^ annually. Output in terms of total biomass (grain, forage, etc.) was 30% greater in non-legumes, which used N to produce dry matter more efficiently than legumes, whereas output of N was greater from legumes. When examined over the crop sequence, the contribution of BNF to the N-balance increased to reach a maximum when the legume fraction was around 0.5 (legume crops were present in half the years). BNF was lower when the legume fraction increased to 0.6–0.8, not because of any feature of the legume, but because the cropping systems in this range were dominated by mixtures of legume and non-legume forages to which inorganic N as fertilizer was normally applied. Forage (e.g., grass and clover), as opposed to grain crops in this range maintained high outputs of biomass and N. In conclusion, BNF through grain and forage legumes has the potential to generate major benefit in terms of reducing or dispensing with the need for mineral N without loss of total output.

## Introduction

Many legumes (plants of the family Fabaceae) form a symbiotic association with types of bacteria that are collectively termed “rhizobia” (Sprent and Sprent, 1990). The rhizobia fix inert atmospheric nitrogen (N) into biologically useful forms within legume root nodules in a process called “biological N fixation” (BNF; Sprent, 2009). This symbiotic association is the largest natural source of the N cycled to sustain natural systems (Vitousek et al., 2002). In addition, BNF by grain and forage legumes plays an important role enriching the pools of soil N for non-N-fixing crops grown after the legumes as part of a strategic cropping sequence or rotation (Bullock, 1992).

Since the discovery of the Haber-Bosch process and the production of anthropogenically produced inorganic N plant fertilizer (Erisman et al., 2008), the ecological and economic dependency of farmed systems on legumes has diminished greatly. For example, the area of European farmland cropped with legumes declined from 11.3 million ha in 1961, to about 3.4 million ha in 2005 (Rochon et al., 2004). This reflects the general preference of farmers for inorganic N-fertilizer which enables the cultivation of non-legume crops commanding higher values in the marketplace and hence greater profit.

Despite the reduction in legume cropping, Europe still relies heavily on legumes for animal feed in the form of imports, especially of soybean (*Glycine max L. Merr*.). European agriculture consumes 25% of the world soybean crop, mainly to sustain livestock production (Steinfeld et al., 2006; UNEP and WHRC, 2007). This trade in soybean encourages tropical deforestation (Simon and Garagorry, 2005; Nepstad et al., 2006). Moreover, the inefficient use of N causes eutrophication and acidification of water, and is responsible for most of the greenhouse gas emissions from farming (Houghton, 1996; Vitousek et al., 1997; EEA, 2007; Sutton et al., 2011).

These environmental concerns, in conjunction with political pressures and market forces that include fuel- and food-insecurity, has made the “sustainable intensification” of cropping systems a main aim of government policy makers (Foresight, 2011). Embodied within this aim is a reappraisal of legume-supported cropping systems, particularly to exploit BNF (Drinkwater et al., 1998), with the aim to mitigate the inefficient use of N and energy in current cropping systems (Hansen, 2000; Hanson et al., 2007). Increasing the proportion of legume crops in a rotation will reduce the use of inorganic fertilizer, increase the proportion of renewable resources of N in global nutrient cycles (Foley et al., 2011; Seufert et al., 2012) and may also decrease the quantity of reactive N lost from the ecosystem.

The impact of the rapid disappearance of legumes from European agriculture remains poorly understood. Agronomic experiments using legumes have neither been coordinated across climatic regions nor analyzed in a consistent manner to quantify the main flows of N in and out of crops and forages. Moreover, in some comparisons, e.g., of organic systems that include legumes against conventional systems that typically do not contain legumes, the productivity of the systems with legumes is generally lower (Seufert et al., 2012). Therefore, there are questions about the ability of legume-based systems to produce output of biomass and N at rates comparable to those of non-legume systems. Accordingly the promotion of legumes in high intensity cropping systems has been difficult to justify. To address this, a comparative “soil-surface N-balance” analysis (Parris, 1998; OECD, 2001), was carried out using data from legume and non-legume supported crop systems at experimental sites that spanned a wide range of cropping systems and pedoclimatic regions across Europe. “Legume inclusion” was defined as the presence of a legume when considering a single cropping-year or the proportion of such years when considering crop sequences, termed “legume fraction”. This approach facilitated a comparative analysis of low-input legume based approaches and conventional approaches. The main questions that are addressed for both single crops and cropping sequences were the degree to which BNF by legumes compensates for the application of mineral N, whether or not legume based rotations contribute to lower surpluses of N, and whether primary production and output as biomass and N are reduced by the inclusion of legumes.

## Materials and methods

### Data sources

Eight case studies of legume based cropping systems originally undertaken as independent trials by the authors were selected. These varied substantially in terms of region, farm system, and experimental design (Table 1). The studies included simple designs of short duration (3 years) to test the benefit of legumes on the yields of following crops, to long-term experiments that monitored the performance of complex legume-based rotations. The systems included arable only, pasture only (grazed, silaged), and mixed systems producing a wide range of legume and non-legume grain, silage and forage products. A number of cropping practices were also included in the systems, namely monocultures, mixtures, intercrops, undersown crops, catch crops and green manures. Together, the eight studies included 29 different main-crops, 59 different main-crop/sub-crop combinations (a sub-crop being defined as an undersown crop or one of the components of an intercrop) and 25 different crop sequences (Table 1).

**Table 1:** **Description of the experimental field trial sites from which N-balance data were sourced**.

**Location**	**Years**	**Production system**	**Sequence length**	**Crops**	**Additional treatments**	**Sequence replication (blocking structure)**	**Example publications**
Aberdeen, Scotland	1991–2006	Organic ley/arable	6	Oats, swede, grass/white clover (3 year ley)		1site/2 blocks/6 plots	Taylor et al., 2006; Watson et al., 2011
Lanna, Saby and Stenstugu Sweden	2005–2009	Conventional ley/arable	6	Spring wheat, winter wheat, oats, barley, grass, winter rape, spring rape, grass/red clover		3site/6blocks/3 sequence/4N fertilizer (on Winter wheat)	Andersson and Milberg, 1996, 1998; Persson et al., 2008; Bergkvist and Båth, 2015
Foulum, Denmark	1997–2008	Organic ley/arable	4	Barley, spring barley, winter wheat, oats, potato, triticale, pea, lupin, faba bean, grass	± manure ± catch crop	1site/2blocks/4plots	Doltra and Olesen, 2013
Foulum Denmark	2006–2008	Organic grass	Variable in 1–4th y of production	Grass, white clover	Each plot split (two halves), with a different N provision N as either slurry (200 kg ha^−1^) or from BNF	1site/20plots	Eriksen et al., 2015
Cordoba, Spain	2008–2012	Conventional arable	2 – Previous crop as treatment	Wheat, sunflower, chickpea, faba bean	Tillage and N fertilizer applied to wheat in sequence	1site/2tillage/ 5previous crop/4N fertilizer rates	Lopez-Bellido et al., 2011
Edinburgh Scotland	2006–2008	Conventional arable without fertilizer	3	Barley, pea/barley, barley/white clover, oat, grass		1site/3blocks/4plots	Pappa et al., 2012
Müncheberg Germany	1995–2006	Organic arable	12	alfalfa, blue lupin, red clover/grass, oat, pea, potato, spring barley, silage maize, spring wheat, triticale, grass, winter wheat, yellow lupin		1 site/8 fields/8plots per field	Bachinger and Reining, 2009; Bloch et al., 2015
San Marco Argentano Italy	2003–2005	Conventional arable	1	Pea, barley, pea/barley	Sowing density	1site/4blocks/ 4treatments	Scalise et al., 2015

### N balance calculations

A soil-surface N-balance analysis was performed in which N inputs and outputs were measured or estimated to allow calculation of N surplus (N input − N output) and N use efficiency (*NUE*, N output / N input; OECD, 2001, 2008) for each crop combination for each year of a crop sequence—defined here as a “*crop-year*.” The input and output data were measured or estimated at the scale of the experimental plot, independently for each plot replicate (where these were present). An N balance for the crop sequence was calculated for each experimental plot by summing the annual balances across the sequence. This sum was then divided by the length in years of the crop-sequence to produce an annual average crop sequence N balance, thereby accounting for the variation in the duration of the crop sequences.

#### N inputs

##### N application as inorganic or organic fertilizer

All experiments reported the application rates for inorganic N (kg N ha^−1^). Organic manure was added to a number of plots and in various forms: solid or slurry, cattle, pig or poultry. The manure application rate (kg ha^−1^) was corrected for dry weight where necessary and multiplied by the total N concentration of the manure to give the *N-in* (kg N ha^−1^). Where manure N concentration was not measured, generic values were taken from the UK Department for Environment, Food and Rural Affairs (DEFRA) Fertilizer Manual RB209 (DEFRA, 2010).

##### Seed N

The N derived from sown seed was estimated by multiplying sowing rates (kg ha^−1^) with seed N content (kg N kg^−1^) either reported by the studies or obtained from the literature. When sowing rates were reported as the number of seeds (m^−2^), values were converted to kg ha^−1^ on the basis of thousand seed weight values obtained from the literature.

##### Biological nitrogen fixation (BNF)

The method of Korsaeth and Eltun (2000) allowed BNF to be estimated in the absence of direct measurement using factors and relations obtained from the literature (Equation 1). This approach was considered sufficiently general that it could be applied to the full range of cropping systems and legume types for the available data.

Adapting this method to the calculation of annual BNF, the N fixed (*N*_*fix*_) by each of the *i* legumes present within a stand over each of the *j* harvests, which in the case of silage might include several cuts in 1 year is:

(1) $$\begin{array}{l} N_{fix}\;=\;\sum_{i}\sum_{j}L_{ij}N_{leg_{ij}}F_{j}R_{j} \end{array}$$

where *L* is the above-ground biomass of the harvested legumes (kg ha^−1^), *N*_*leg*_ is an assumed fraction for crop N composition, *F* is proportion of legume N derived from air by BNF, and *R* is the ratio of shoot to total plant biomass for each legume. In addition, *F* is assumed to be sensitive to the amount of inorganic N in the soil. The proportion of N present within a plant derived from BNF is greatest in the absence of any inorganic N and declines in a linear way with the increasing application of inorganic N, i.e.,

(2) $$\begin{array}{l} F_{j}\;=\;F_{max}\;-\;a_{j}N_{inorganic} \end{array}$$

Here it is assumed that inorganic N (kg N ha^−1^) can be derived from chemical fertilizer and the inorganic component in organic fertilizers.

##### Outputs

Nitrogen output was estimated on the basis of the amount of N removed from the system in crop biomass and livestock. Losses of N to the atmosphere and through leaching were not available in all cases and hence were not accounted for.

##### Harvest of crop

Depending on the experimental treatment, harvested material was either removed from the plots or left to be incorporated at a later stage. In most cases, a separate biomass measurement was reported for each component of this output; otherwise, a total biomass and relative proportion of each component was reported from which output biomass was then calculated. The amount of N removed from the plot at harvest was calculated using the N fraction of each component. Where the N fraction was not measured directly, estimates were taken from the literature.

##### Livestock

Livestock were assumed to contribute to N losses by the consumption and assimilation of N through grazing and the subsequent removal of the animals from the plot or field. Where it took place, grazing was by sheep and was reported in terms of livestock unit grazing days (LUGD) from which N consumption was calculated as follows:

(3) $$\begin{array}{l} N_{consumption}=\;LUGD\;\times \;W_{LUGD}\;\times \;C\;\times \;N_{forage} \end{array}$$

Here *LUGD* is converted to a weight equivalent given a livestock unit weight (*W*_*LUGD*_) of 650 kg (c.f. DEFRA, 2011), and multiplied by a consumption rate (*C*) in sheep of 3% body-mass *per* day and the fractional composition of N in the forage crops (*N*_*forage*_). To account for the return of N to the field through excretion, it was assumed that 10% of consumed N was assimilated and retained by the sheep (IPCC, 2006; Table 10.22).

##### Productivity

The biomass (kg ha^−1^) of crop output, including grain, straw and silage, was derived for each year of each crop sequence to provide a measure of productivity. Biomass output efficiency (*BOE*) was calculated as the biomass of crop output achieved for each kilogram of N input.

### Data analysis

The influence of legume cropping on N balances was analyzed in terms of N input (*N-in*), the contributions to this from the application of fertilizer (N-*fert*) and from BNF (N-*fix*), N output (N-*out*), N surplus (N-*surp*), the difference between N-*in* and N*-out*, and N use efficiency (*NUE*; N-*out / N-in*; OECD, 2008). The analysis also considered productivity in terms of the variables Biomass Output and Biomass Output Efficiency (*BO* and *BOE*, respectively). Together these are referred to as the “N balance variables.” The analysis was conducted in 2 parts, the first addressing the annual N balance variables for *crop-year* and second the annual average crop-sequence N balances.

#### Annual N balance

The frequency distributions of the N balance variables across datasets, plots and years were positively skewed and included a number of zero values (with the exception of N-*surp*). A log plus constant transformation of the N balance variables resulted in a bimodal distribution, indicating that the experimental plots could be considered as falling into two populations. Firstly, those for which N-*in* or N-*out* were zero or very small and secondly, those with a positive N-*in* and N-*out* (Table 2). Thresholds to discriminate between these populations were selected by examination of their bimodal frequency distributions. Whether a plot received an input or produced an output (i.e., positive N-*in* or N-*out*) was largely determined by the chosen crop management practices e.g., whether a legume was included, fertilizer applied, or silage removed after cutting. Therefore, these categorical states do not lend themselves to being considered as random variables suitable for statistical analysis. However, the proportion of observations falling into these categorical states has been used to describe the crop management conditions under which the experiments were carried out.

**Table 2:** The number of experimental plots (crops y^**−1**^), for which N balance variables were either low or high for set “value ranges” (kg N ha^**−1**^)^*^.

**Value range (kg N ha^−1^)**	**N fertilizer**		**N fixed**		**N input**		N output^*^,^†^		Biomass output^*^	
	**[0 and 2]**	**[30 and 345]**	**0**	***≥1***	**[0 and 25.5]**	**>25.5**	**0**	***≥1***	**0**	***≥1***
Grain legume	99	0	1	98	1	98	0	99	0	99
Grain mix	36	2	0	38	0	38	0	38	0	38
Grain non-legume	393	730	1123	0	389	734	22	1097	22	1101
Forage mix	352	180	141	376	138	379	83	434	184	348
Forage non-legume	44	102	124	22	36	110	0	146	0	146
Grain legume + forage	73	1	0	74	0	74	0	74	0	74
Grain + forage legume	86	152	203	30	66	167	0	232	0	237

*The sum of the number of low and high experimental plots is not consistent across N balance variables in some cases due to missing observations*.

* *Zero values reported for N- and Biomass-output variables reflect the fact that there was no removal of crop material from the system*.

† *Output N values do not include N losses due to leaching or denitrification, as stated in the Materials and Methods*.

Data from the low value populations as defined (see Table 2) were excluded from further analysis. The N balance variables, comprised of high value observations only, were then treated as continuous variables and analyzed using linear mixed-effects models, following a natural log + constant transformation, with the exception of N-*surp* which was not transformed. In modeling all N balance variables, *site* and *plot* variables were included as random terms to account for consistent differences between experiments and between the experimental plots within each experiment. The hypothesis that there was no difference in the N balance variables between legume and non-legume crops was tested by a likelihood ratio test of competing models. For this analysis, the large number of different combinations of legumes, non-legumes, grain crops and forages was condensed into five comparisons, each consisting of a legume and a non-legume: (1) grain legume (e.g., faba bean) vs. grain non-legume (e.g., maize); (2) legume and non-legume grain crops (e.g., pea and barley), referred to as “grain mix” vs. grain non-legume (e.g., maize); (3) legume and non-legume forage crops (e.g., grass and clover), referred to as “forage mix” vs. forage non-legume (e.g., grass); (4) grain legume and non-legume forage (e.g., faba bean and rye) vs. grain and forage non-legume (e.g., oat and rye); and, (5) grain and forage legume (e.g., barley and clover) vs. grain and forage non-legume (e.g., barley and grass). Where different types of crop or forage were indicated in the above list, they were both grown at some point during the same year; no distinction was made as to whether the crops were grown in sequence or as intercrops.

Where appropriate, data were back transformed following analysis and values reported in kg ha^−1^ to facilitate comparison with other data sources.

#### Crop sequence N balance

To evaluate the role of legumes within a crop sequence, the yearly average of each N balance variable was calculated for each crop sequence of 4 or more years. In addition, the proportion of years in which a legume crop was grown was calculated, as was the proportion of grain, forage and grain/forage combinations.

The relation between each crop sequence N balance variable and the proportion of legume cropping in a crop sequence was examined and the significance tested by fitting a generalized additive model (GAM) to the data assuming Gaussian errors and an identity link-function. Each N balance variable was modeled independently as a smooth function of the proportion of years of a crop sequence that included legumes. The significance of the smooth term was then tested using Analysis of Deviance to compare the model against a second model from which the smooth term was excluded.

## Results

### N balance: crops

#### Broad comparison with and without fertilizer

**Figure 1:**
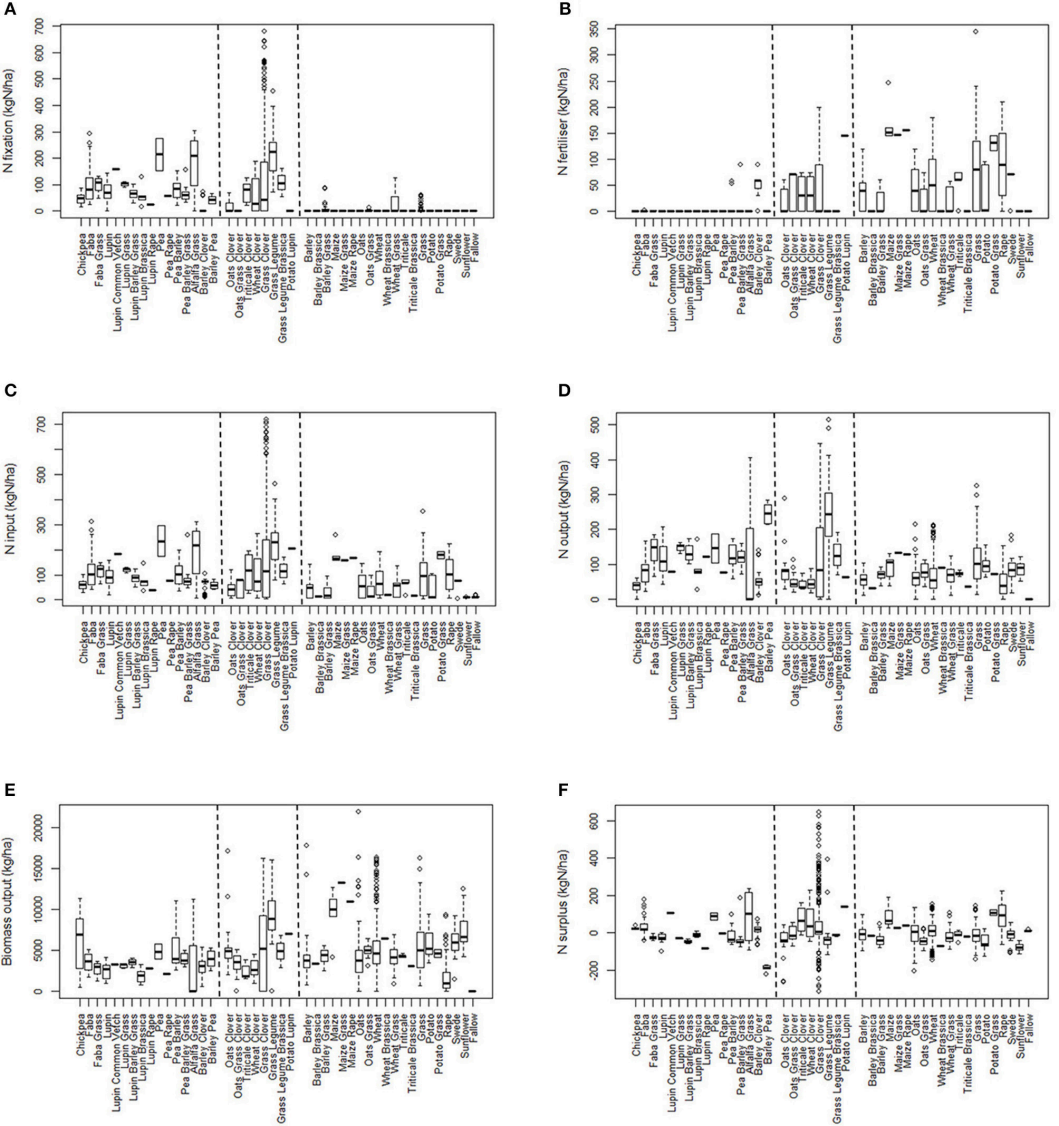
**Box and whisker plots showing the levels of: (A)**, N-*fix*; **(B)**, N-*fert*; **(C)**, N-*in*; **(D)**, N-*out*; **(E)**, N-*surp* (kg N ha^−1^); and, **(F)**, Biomass output (kg ha^−1^) for all crop combinations. The first block of crops on the left side of the x-axis consists of legumes that received no or very little fertilizer; the second block, legume and non-legume mixtures that generally received fertilizer; and the third block, non-legumes.

The crop-types are first arranged into three broad groupings defined by whether they included legumes and/or received mineral fertilizer (Figures 1A–F). The “legume” group (left hand side of each figure), comprised legumes either alone or in combination with another crop, and with a few exceptions did not receive mineral fertilizer in the year in question. The “legume mix + fertilizer” group (center in each part of Figure 1) included mixtures of legumes and non-legumes, for example where the legume is undersown, and where the crop in that year received mineral fertilizer. The “non-legume” group comprised single and mixed crops such as cereal, potato and grass which generally received mineral fertilizer.

In interpreting these data, only N-*fert* is based on measured inputs during the year, whereas the contribution from N-*fix* can only be accounted when biomass was removed from the field during the year in question. All the apparent BNF is accounted for in that 1 year if biomass had been accumulating for two or more years before its removal. This facet of the calculation is responsible for some of the very high values (475–700 kg N ha^−1^ y^−1^) of N input for grass-clover and grass-legume mixtures (Figure 1).

#### Specific comparison of different crop-types

As defined in Materials and Methods, the data allowed specific comparisons between five types of crop and forage with and without a legume (Table 3). Biomass production averaged in these groupings ranged from 2.4 to 5.5 t ha^−1^ (back-transformed means). In all but one of the comparisons, the non-legume class produced significantly more biomass than the respective legume class. Only “forage non-legume” produced less than its comparator. Biomass across the five non-legume classes was on average 1.29-fold that of the legume (based on back-transformed values). In contrast, N-*out* differed in the opposite direction to that of biomass itself, being greater in the legume than the non-legume in four out of five comparisons (the exception being the grain + forage in mixed rotations; last 3 rows of Table 3). In terms of back-transformed means, the range of N-*out* was 55 to 115 kg ha^−1^. The greater N but smaller biomass values in the legume component reflects the generally higher %N in legume tissue compared to non-legume tissue.

**Table 3:** **Comparison of N budget variables between the five categories (see column 1): grain and forage, and legume and non-legume crop combinations for those plots for which the N budget variables were high (see Table 2), “na,” denotes “non-applicable” comparisons**.

	**N fertilizer (kg N ha^−1^)**	**N fixed (kg N ha^−1^)**	**N input (kg N ha^−1^)**	**N output (kg N ha^−1^)**	**N surplus (kg N ha^−1^) (N-*out* > 0)**	**Biomass output (kg N ha^−1^)**	***NUE* (*N-out* > 0)**	***BOE***
Grain legume	0.36 ± 0.00 (1.43)	4.20 ± 0.06 (66.7)	4.68 ± 0.09 (107.8)	4.32 ± 0.11 (75.2)	6.98 ± 17.74	7.79 ± 0.20 (2416.3)	0.12 ± 0.36 (1.13)	3.57 ± 0.43 (35.5)
Grain non-legume	4.30 ± 0.03 (73.7)	na	4.44 ± 0.08 (84.8)	4.06 ± 0.10 (58.0)	−10.08 ± 16.93	8.36 ± 0.19 (4272.7)	0.75 ± 0.35 (2.12)	5.03 ± 0.42 (152.9)
*Test*	*na*	*na*	*24.71 <0.0001*	*14.06 < 0.0001*	*7.96 < 0.0048*	*56.26 < 0.0001*	*38.23 < 0.0001*	*168.3664 < 0.0001*
Grain mix	4.01 ± 0.04 (55.1)	4.19 ± 0.08 (66.0)	4.73 ± 0.13 (113.3)	4.70 ± 0.13 (109.9)	−24.76 ± 19.84	8.10 ± 0.22 (3294.5)	0.15 ± 0.37 (1.16)	3.45 ± 0.43 (31.5)
Grain non-legume	4.30 ± 0.03 (73.7)	na	4.33 ± 0.13 (75.9)	4.06 ± 0.09 (58.0)	−12.74 ± 18.27	8.37 ± 0.19 (4315.6)	0.74 ± 0.34 (2.10)	5.04 ± 0.41 (154.5)
*Test*	*na*	*na*	*61.0749 < 0.0001*	*41.15 < 0.0001*	*1.78 < 0.1820*	*4.45 < 0.0349*	*16.15 < 0.0001*	*78.07 < 0.0001*
Forage mix	4.39 ± 0.18 (80.6)	4.75 ± 0.26 (115.6)	5.16 ± 0.14 (174.2)	4.75 ± 0.26 (115.6)	−20.48 ± 20.80	8.61 ± 0.21 (5486.2)	0.66 ± 0.36 (1.93)	4.29 ± 0.28 (73.0)
Forage non-legume	4.95 ± 0.17 (141.2)	2.27 ± 0.33 (9.7)	4.93 ± 0.15 (138.4)	4.34 ± 0.26 (76.7)	28.04 ± 21.16	8.44 ± 0.21 (4628.6)	0.20 ± 0.36 (1.22)	4.16 ± 0.28 (64.1)
*Test*	*19.77 < 0.0001*	*108.92 < 0.0001*	*8.30 < 0.0040*	*14.29 < 0.0001*	*33.81 < 0.0001*	*8.31 < 0.0039*	*24.867 < 0.0001*	*1.76 < 0.1851*
Grain legume + forage	0.36 ± 0.00 (1.40)	4.23 ± 0.10 (68.7)	4.47 ± 0.04 (87.4)	4.66 ± 0.15 (105.6)	−34.28 ± 4.27	8.00 ± 0.12 (2981.0)	0.34 ± 0.09 (1.40)	3.65 ± 0.09 (38.5)
Grain + forage	*na*	3.14 ± 0.13 (23.0)	4.17 ± 0.04 (64.74)	4.04 ± 0.15 (56.8)	−28.64 ± 3.31	8.22 ± 0.12 (3714.5)	0.83 ± 0.07 (2.29)	4.96 ± 0.07 (142.6)
*Test*	*na*	*37.24 < 0.0001*	*29.22 < 0.0001*	*120.21 < 0.0001*	*1.10 < 0.2940*	*22.67 < 0.0001*	*18.07 < 0.0001*	*98.91 < 0.0001*
Grain + forage legume	4.13 ± 0.04 (67.2)	3.48 ± 0.58 (32.5)	4.29 ± 0.08 (73.0)	4.01 ± 0.17 (55.1)	−8.66 ± 19.99	8.12 ± 0.11 (3361.0)	0.19 ± 0.19 (1.21)	4.32 ± 0.18 (75.2)
Grain + forage	3.95 ± 0.05 (49.4)	2.48 ± 0.60 (11.9)	4.03 ± 0.09 (56.3)	4.25 ± 0.18 (70.1)	−57.78 ± 20.65	8.35 ± 0.12 (4230.2)	0.92 ± 0.22 (2.51)	5.05 ± 0.21 (156.0)
*Test*	*14.69 < 0.0001*	*8.68 < 0.0032*	*17.52 < 0.0001*	*17.11 < 0.0001*	*45.03 < 0.0001*	*14.81 < 0.0001*	*26.84 < 0.0001*	*26.12 < 0.0001*

*F statistics and probability values obtained from analysis of variance are given in italics, non-italicized text are means ± standard errors. All data are log-transformed with the exception of N surplus, back transformed mean values are included in brackets. “NUE,” denotes nutrient use efficiency, and “BOE,” biomass output efficiency*.

Whether or not the majority was provided by N-*fert* or N-*fix*, N*-in* was greater in the legume class in all five comparisons. In the cases where N-*fix* was estimated in a non-legume crop, this was due to BNF by legumes that were neither sown nor intended to be in the plot (e.g., legume forages as volunteer weeds). N*-surp* varied substantially within and between experiments and experimental plots (see standard errors in Table 3), and for the majority of crop types it cannot be established whether grain crops, legume or non-legume, consistently produce either a surplus or deficit of N.

The two “efficiencies” *BOE* and *NUE*, were generally consistent with what might be expected from the other N-balance variables. In all cases, the non-legume, with its generally higher biomass output and lower N*-in*, achieved higher *NUE* and *BOE*. However, the output of biomass and N by the legumes was achieved in the absence, or reduced application of, fertilizer and hence was highly efficient in terms of biomass *per* unit N applied as fertilizer.

Unlike grain legumes which were grown alone, forage legumes were only grown in combination with non-legumes, either grain or forage. The combination of both legume and non-legume species blurred the distinction between these crop types, such that substantial amounts of N-*fert* were applied to legume-containing forages and also BNF was estimated as positive in non-legume crops due to “contamination” by legumes grown previously. Among the three forage comparisons (Table 3) the vegetative crops (third comparison) whether legume (e.g., clover and grass), or non-legume (e.g., grass), gave the highest N-*in*, N-*out* and biomass.

### N balance: crop sequence

**Figure 2:**
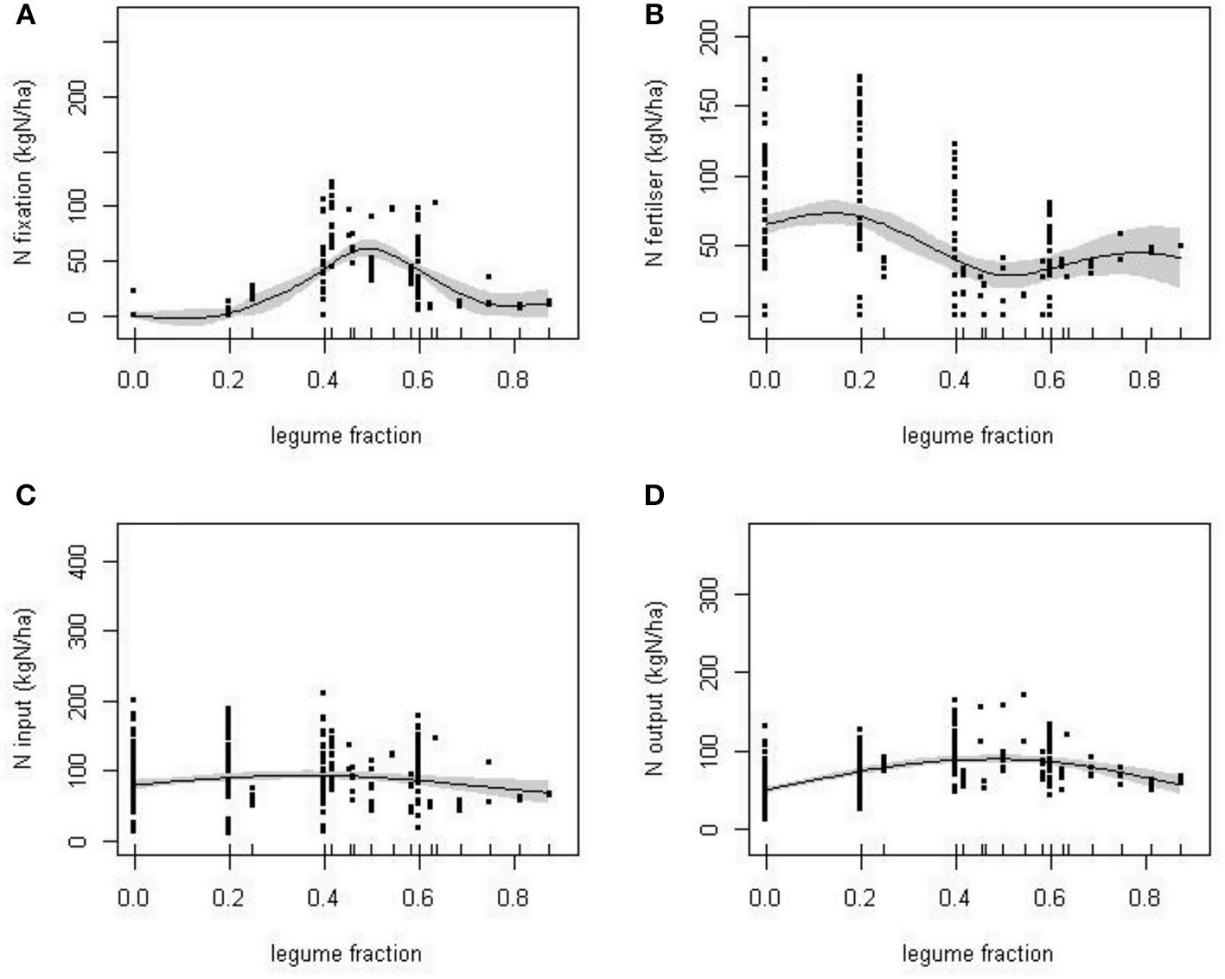
**The response of: (A)**, BNF; **(B)**, N fertilizer input; **(C)**, N input; and, **(D)**, N output to the proportion of the experimental cropping sequences by year in which legumes were cultivated (legume fraction). Solid lines are estimated smooth functions obtained from fitted GAMs with standard errors given by the shaded areas.

The average annual rate of *N-fix* did not increase monotonically with the proportion of legume crops but peaked at an intermediate proportion of legume crops (Figure 2A). To test the significance of this pattern, a generalized additive model (GAM) was fitted to the data in which average BNF was modeled as a smooth function of the proportion of years of a crop sequence that included legumes, excluding the 100% legume sequences. The smooth term was significant [F_(4.28, 290)_ = 38.7, *P* < 0.001] indicating a peak in BNF of 50–100 kg ha^−1^ (Figure 2A).

Though the variation in the average rate of N application between crop sequences was, to a large extent, independent of legume cropping, there was a significant reduction in *N-fert* at approximately 50% legume fraction [F_(2.66, 291_ = 8.83, *P* < 0.001; Figure 2B]. The trade-off between *N-fert* and *N-fix*, the two major sources of N, acts to minimize the sensitivity of total *N-in* to the extent of legume cropping (Figure 2C), although *N-in* was slightly elevated at intermediate levels of legume use [F_(0.92, 293)_ = 10.30, *P* = 0.002]. The trend in N output (Figure 2D) is similar to that of *N-fix* and *N-in* including a peak at intermediate levels of legume cropping [F_(1.99, 292)_ = 29.11, *P* < 0.001].

**Figure 3:**
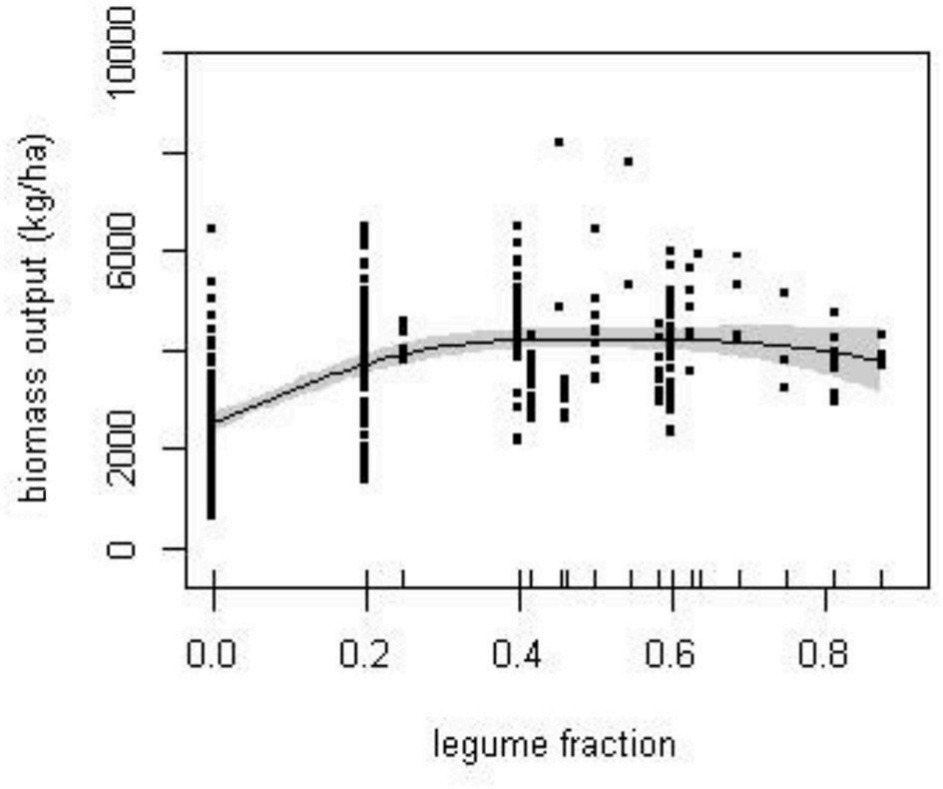
**The response of biomass production to the proportion of the experimental cropping sequences by year in which legumes were cultivated (legume fraction)**. Solid line is the estimated smooth function obtained from fitted GAM with standard errors given by the shaded area.

The trade-off between *N-fix* and *N-fert* is, in part, to be expected as a consequence of the negative relationship between N application and BNF assumed in the calculation of *N-fix* (Equation 2). However, the increase in legume fraction is also associated with a shift in cropping system. For example, many sequences with a legume fraction above 0.6 were of a similar mixed cropping system dominated by a grazed grass-clover ley. This is a largely vegetative combination, producing higher biomass and N output than other crops, but habitually given N fertilizer even though legumes were present in most years. The various compensations between *N-fix* and *N-fert* resulted in a broadly similar biomass output over a wide range of legume fractions (Figure 3), from around 25% legume inclusion.

## Discussion

### Comparative performance of legume and non-legume crops

The main questions to be answered by this analysis are whether BNF by legumes compensates for reduced input of mineral fertilizer and whether having a legume in a crop sequence reduces overall productivity. When analyzed by *crop-year*, the two measured variables, biomass and N fertilizer, were not tightly coupled i.e., large quantities of biomass were produced in the absence of fertilizer. Legumes had compensated by providing the N requirements in treatments that received no or reduced fertilizer. In all five assessments by crop-type (Table 3), the legume generated a higher *N-in* through a combination of measured *N-fert* and estimated *N-fix*, than the non-legume comparator.

The observation that a mixture of legumes and non-legumes increases productivity is consistent with the observations of a large European study that analyzed the productivity of grass swards with differing proportions of clover (Suter et al., 2015). They suggested that competition for N, N transfer, and niche complementarity could explain the synergies associated with such mixtures. In our study the effects of the legumes on productivity (here measured as output) was more complex and depended on how productivity was defined. Non-legumes, such as maize and grass, produced about 1.3-fold the biomass of legumes but about 0.75-fold the N in the output, consistent with the generally higher %N of legumes. This general conclusion was modified in favor of the legume for the one comparison that consisted of all-vegetative crops, namely the forage mix (e.g., clover and grass) and the forage non-legume (e.g., grass), where N-out and biomass were both higher in the grass-legume mixture. The highest biomass and *N-out* among all comparisons were produced by this vegetative forage (all others having some “grain” component). The factor probably responsible for the higher productivity of these forages was the absence of a reduced photosynthetic capacity that normally occurs during reproductive growth, as a result of the requirement of the grain for N, which usually triggers leaf senescence. There might also have been some niche complementarity between the vegetative legume BNF and the non-legume using applied N, resulting in the forage mix having greater output than the comparator (Lüscher et al., 2014).

### N-balance approach

The N-balance approach has the advantage that it allows systematic comparison of a wide range of disparate studies that could otherwise not be pooled for analysis, but inconsistencies can enter the analysis since no account is taken of internal cycling of N. Potential carry-over effects between years, e.g., through uptake from N pools in soil, “catch” crops that were purposely plowed and did not contribute directly to output, and persistence of legumes as volunteer weeds can all affect single-year estimates of N-balance variables. However, the examination of the balance over the sequence should negate, or at least reduce, these potential complications. When considered over the sequence, both N*-out* (Figure 2) and to a lesser extent biomass (Figure 3) showed a broad maximum at legume fraction between 0.3 and 0.7, within which highest values were between 0.4 and 0.6. In contrast, the two components of *N-in* varied systematically and generally in opposition in their contribution. Together these trends confirm the previous conclusion based on *crop-year* that BNF compensated for reduced mineral fertilizer in generating biomass.

Between legume fractions of 0.6 and 0.8, the contribution of *N-fix* decreased. Most sequences in this range were forages that typically included legumes, either as intercrops or in relay, but were also given fertilizer, even in years when the legumes were present, such that *N-fix* was estimated as a smaller input than it was from grain legumes. These sequences nevertheless, through a combination of *N-fert* and *N-fix*, maintained high *N-in*, and *N-out* and biomass. Therefore, the presence of legumes did not cause any systematic reduction in total output when assessed over the cropping sequence (Figure 3).

### Opportunity for increasing BNF and other properties of legumes in multifunctional systems

The rates of output and *N-fix* were measured here in experimental systems, and with rotations, that included those designed for productivity in low-input or organic conditions. Nevertheless, N-balance variables and yield were within the range of values commonly found in high input cropping. For example, the combined inputs of N by BNF and fertilizer, *N-in*, ranged from 56 to 174 kg ha^−1^ for the legumes, while mean *N-fix* ranged from 32 to 115 kg ha^−1^ (Table 3, back-transformed means). In comparison, the typical mineral N input in conventional high input cropping in Europe is around 100 kg ha^−1^ for a spring cereal and 200 kg ha^−1^ for a winter cereal or oilseed (Fertiliser Practice, 2014). The most direct comparator for high input cereals is that of grain legume crops (the first comparison at the top of Table 3). Here, *N-in* for the grain legume was 108 kg ha^−1^ and biomass output 2.4 t ha^−1^; while *N-in* for the non-legume grain was 85 kg ha^−1^ and biomass output 4.3 t ha^−1^. Given that grain yields for high-intensity cereals are typically 4–8 t ha^−1^, the lower value being for spring crops, (Sylvester-Bradley and Kindred, 2009) the non-legume grains in this study are comparable with general yields, while the legume grains show the typical higher N and lower biomass in yield.

Higher values of *N-fix* than those recorded here should be feasible. While the agronomy underpinning the trials was advanced by conventional standards, no particular attempt was made to maximize the amount of *N-fix*. Indeed, the common inclusion of *N-fert* in many of the sequences would have had the opposite effect. Moreover, values of *N-fix* estimated here were for current varieties that have been bred largely for conventional high-input cropping systems where there is generally much residual N. The potential for improving BNF within existing species and varieties of grain legume needs to be considered. For example, faba bean has been shown to have a significantly greater effect than other legumes on the yield of subsequent cereal grain crops (Wani et al., 1991; Hauggaard-Nielsen et al., 2009, 2012). Therefore, legume breeding should aim for higher levels of performance directed at low-input environments, cropping-systems focused on energy as well as food and fodder production (Porter et al., 2009; Jensen et al., 2012) and in both mixed cropping or intercropping (Brooker et al., 2014).

Legumes may have a range of benefits other than through BNF and potential as a green manure. Some degree of persistence by living legume plants to the next phase of the cropping-sequence was found in a number of plots and while there may be risks of such carry-over (Driscoll et al., 2014), the production benefits from the legume forages and grains that persist into subsequent crops need to be quantified and exploited. Below-ground phenology will also be important. Faba bean presents extensive tap roots capable of reaching deep into the soil profile, improving soil structure and benefiting the rooting profile of crops which follow them in the rotation (Rochester et al., 1998, 2001). In relation to this, the benefit of grain legumes to the yield of the next crop is greater than the benefit brought by carry-over of N in the soil. Some other factors are influencing yield positively, implying a cascade effect to other soil- and system-processes beyond enhanced soil N status (Danso and Papastylianou, 1992; Ehrmann and Ritz, 2014). This pre-crop benefit of grain legumes can lead to yield increases of up to 1.6 t ha^−1^ in subsequent cereals (Preissel et al., 2015). This is consistent with our own findings which assessed the pre-crop benefits of legumes at the rotational level, and which also suggest higher non-legume yield potential may be achieved in years after legumes. This reflects the higher N inputs achieved by legume supported rotations.

However, in tackling problems such as reducing N losses from agriculture, legumes should also have a role but as part of broader systems of management. Greenhouse gas emissions, for example, depend on the complex interaction of several different biotic and abiotic variables (Snyder et al., 2014). While legume supported crop systems may limit N and C losses (Drinkwater et al., 1998, maize-soybean), management per se is a major factor in achieving this (Rees et al., 2013), especially the use of cover crops, both legume and non-legume grown to accumulate N in the soil for potential incorporation by later crops and to reduce GHG emissions (Thorup-Kristensen et al., 2003; Li et al., 2015).

A more critical appraisal of the traits targeted by legumes breeders should consider these wider system benefits (Sinclair and Valdez, 2012). Improvements are feasible e.g. soybean demands manipulation by either agronomy or re-breeding to render it as a net “contributor” of N, as opposed to a net “taker” from the system (Alves et al., 2003; Perez de la Vega et al., 2011; Baddeley et al., 2013). Moreover, the purpose of legumes in mixed forage systems, both examined here and in the wider literature, is not simply to contribute BNF or in-field benefits, but to bring wider system benefits beyond the farm level e.g., as a forage with particular characteristics or through a combination of outputs that optimize biomass and N content (Danso and Papastylianou, 1992; Crews and Peoples, 2004; Foley et al., 2011).

## Conclusions

The data assessed here were generated from very different experimental designs adapted to a wide range of pedoclimatic and socioeconomic conditions and presented a significant challenge to ensure they were harmonized to allow comparative analysis of a range of conventional, low-input and organic approaches. It is possible that a more marked efficiency benefit would have been realized if intensive systems had also been considered in the comparative analysis. Nevertheless, a model is suggested which proposes that optimum efficiency is achieved with an approximately equal balance of forage and grain legumes such that the legume fraction, the proportion of rotation years in which a legume is cropped, is around 0.4–0.6, with the legume component often accommodated as an intercrop.

Mathematical modeling studies have indicated that legume based crop rotations can deliver environmental and economic benefits (Reckling et al., 2016). However, the optimal rate of legume inclusion, such as the high inclusion levels reported here, should be tested and refined empirically with respect to the specific pedoclimatic and socio-economic contexts for those regions in which they are cultivated. Such tests should assume rotations which accommodate intelligent design principles which include agronomic approaches that mimic those assessed here for low-input, organic and often mixed cropping systems. Additionally, major challenges will remain, such as ensuring that losses though leaching and greenhouse gases are minimized. It is also important that effective integrated pest management strategies for legume supported systems are also developed.

## Author contributions

The approach was conceived jointly by GB and PI. The original data files were verified and collated in an open-access (on request) Access^TM^ database by MY. GB, PI and MY, jointly coordinated to collate and harmonized the datasets in a standard data-template, specifically designed for the soil-surface N balance analysis and to facilitate comparisons between trials. Data analysis and initial interpretation of the results was led and carried out by GB solely. GB, PI, MY, GS, RR and EJ played key roles in developing the final interpretation of the results, and finalizing the discussion. All other authors contributed the original datasets, and contributed to the preparation of the manuscript by recommending supporting scientific references and providing critical comment.

## Funding

This research was supported with funding from EU-FP7 project Legume Futures (http://www.legumefutures.eu), under grant agreement number 245216 CP-FP. The James Hutton Institute and the Scottish Rural Colleges and the associated staff are also supported by the Scottish Government.

### Conflict of interest statement

The authors declare that the research was conducted in the absence of any commercial or financial relationships that could be construed as a potential conflict of interest. The reviewer SDSK and handling Editor declared their shared affiliation, and the handling Editor states that the process nevertheless met the standards of a fair and objective review.
